# The antipyretic effectiveness of dipyrone in the intensive care unit: A retrospective cohort study

**DOI:** 10.1371/journal.pone.0264440

**Published:** 2022-03-10

**Authors:** Hans-Jörg Gillmann, Jessica Reichart, Andreas Leffler, Thomas Stueber

**Affiliations:** Department of Anaesthesiology and Intensive Care Medicine, Hannover Medical School, Hannover, Germany; The University of Mississippi Medical Center, UNITED STATES

## Abstract

**Introduction:**

Dipyrone (metamizol) is regularly used in critical care for pain and fever treatment, especially in Germany and Spain. However, indication for antipyretic therapy in critically ill patients is currently unclear and data for both the risk and benefit of dipyrone treatment in the intensive care environment are scarce. We hypothesized that antipyretic efficiency of dipyrone would not exceed antipyretic efficiency of acetaminophen. We therefore aimed to compare temperature courses in critically ill patients receiving either intravenous dipyrone, acetaminophen or no antipyretic medication.

**Material and methods:**

We included 937 intensive care unit (ICU) patients with body temperature recordings of at least 37.5°C. We investigated temperature decrease associated with dipyrone or acetaminophen and additionally compared it to an untreated control group.

**Results:**

Within the eight-hour study interval, maximum body temperature decrease in patients without antipyretic medication was -0.6°C (IQR: -1.0 to -0.4°C; n = 315). Maximal decrease in body temperature was higher both with dipyrone (-0.8°C (IQR: -1.2 to -0.4°C); p = 0.016; n = 341) and acetaminophen (-0.9°C (IQR: -1.6 to -0.6°C); p<0.001; n = 71), but did not differ between dipyrone and acetaminophen (p = 0.066). As compared to untreated patients, dipyrone only led to a marginal additional decrease in body temperature of only -0.1°C. Maximum of antipyretic effectiveness was reached four hours after administration.

**Conclusion:**

Antipyretic effectiveness of dipyrone in ICU patients may be overestimated. Given the lack of prospective data, clinical evidence for antipyretic dipyrone therapy in the ICU is insufficient and warrants further critical evaluation.

## Introduction

Dipyrone (metamizol) is regularly used in critical care for treatment of pain and fever. While dipyrone has been withdrawn from the market for example in the United States of America, the United Kingdom and Japan, especially in Germany and Spain dipyrone is frequently used during perioperative treatments [1]. However, data for both the risk and benefit of dipyrone treatment in the intensive care environment are scarce [2]. According to a recent meta-analysis the short-term treatment with dipyrone may be save, but several hematologic, renal und liver side effects are discussed in the literature [3–6]. Having said that, a recent survey of our group indicated that dipyrone and acetaminophen are the most commonly used drugs for antipyretic treatment in critical care [7]. The exact antipyretic mechanisms of both acetaminophen and dipyrone are currently unknown. Acetaminophen may induce antipyretic effects by direct inhibition of cyclooxygenase or by its metabolite N-Arachidonoylphenolamin (AM404) via transient receptor potential vanilloid-1 receptors (TRPV1) [8]. Dipyrone may also induce antipyretic effects by direct cyclooxygenase inhibition or via interaction of its metabolites with TRPV1 and transient receptor potential ankyrin A1 receptors (TRPA1) [9].

Indication for antipyretic therapy in critically ill patients is currently unclear, and even definition of fever in critically ill patients is heterogeneous [10, 11]. In the so-called HEAT trial, treatment of fever with acetaminophen did not reduce the number of ICU free days [12]. Remarkably, the effect of acetaminophen on the mean daily peak and mean daily average body temperature was only modest with an absolute temperature difference of around -0.25°C compared to placebo. Comparable data for the antipyretic effect of dipyrone in the critical care environment are lacking. In a small observational study, dipyrone decreased mean temperature by 1.1°C on average as compared to acetaminophen by 0.9°C 120 min after infusion [13]. In another small observational study in patients with subarachnoid hemorrhage, the maximal decrease of body core temperature after dipyrone was 1.0°C [14]. Unfortunately, both studies did not include an untreated control group with fever.

Taken together, dipyrone is often used for antipyretic therapy in critical care. However, safety data for antipyretic therapy with dipyrone are lacking and current evidence for the antipyretic effectiveness in critically ill patients are based on scarce and small studies lacking untreated control groups. We hypothesized that antipyretic efficiency of dipyrone would not exceed antipyretic efficiency of acetaminophen. We therefore aimed to compare temperature courses in critically ill patients receiving either intravenous dipyrone, acetaminophen or no antipyretic medication.

## Methods

### Study design and population

Ethical approval for this study (Ethical Committee N° 9218_BO_K_2020) was provided by Hannover Medical School Ethics Committee, Hannover, Germany (Chairperson Prof. S. Engeli) on 21th of July 2020. This retrospective study was designed to explore antipyretic efficiency of intravenous dipyrone treatment. We intended to compare dipyrone treated patients both with (1) acetaminophen treated patients and (2) additionally with a group of ICU patients without dedicated antipyretic treatment. Indications and cutoffs for antipyretic treatment are based on a broad and in part contradictory range of evidence [10, 11, 15, 16]. In some patient groups, antipyretic treatment is initiated prior to reaching consented but various temperature levels in order to prevent patients from elevated temperature episodes [17, 18]. For this retrospective study, we therefore included patients admitted to the anesthesia intensive care unit (ICU, Department of Anesthesia and Intensive Care Medicine, Hannover Medical School) from January 2015 until July 2020 were screened for temperature registrations of 37.5°C or higher. For dipyrone and acetaminophen treated patients, body temperature at the time point of antipyretic drug administration was defined as the time reference point (i.e. 0h). Representative body temperatures were extracted one hour prior as well as two, four, six and eight hours after antipyretic medication. In patients without antipyretic treatment, spontaneous temperature courses were extracted with the reference time point (i.e. 0h) at the maximum temperature recorded during the ICU stay. We chose maximum temperature as the reference time point (1) to achieve an investigator-independent data extraction and (2) to compare dipyrone and acetaminophen treatment with maximum temperature decrease in untreated critically ill patients. This approach allowed us to estimate the minimum additional temperature decreasing effect associated with dipyrone or acetaminophen as compared to spontaneous temperature courses without dedicated antipyretic medication.

We have characterized the study ICU and the treated patient collective before [16]. In summary, the anesthesia ICU is one of six independent surgical intensive care units at Hannover Medical School as a tertiary referral hospital. The ICU consists of 14 beds and provides care for a mixed surgical patient collective, interventional cardiology and neurology patients (approximately 90% post-surgery or post-interventional cases). A standard operating procedure (SOP) for postoperative pain management recommended dipyrone as the non-opioid analgesic of first choice, but there was no dedicated SOP for fever management or universal temperature treatment thresholds. The intensive care physician in charge was responsible for prescription of postoperative pain therapy, temperature management, choice of drugs and type of application.

### Inclusion and exclusion criteria

To reproduce the current wide range of fever definitions and also to include patients where avoidance of fever episodes might be intended, we included patients with a peak temperature measurement of at least 37.5°C. Additional inclusion criteria were (1) continuous temperature measurement with automated documentation (bloodstream or urinary bladder temperature registrations), (2) with an ICU treatment duration of at least 24h and (3) aged 18 years or older. Patients were excluded if (1) admitted repeatedly (only the most recent stay in the study interval was evaluated), (2) only oral antipyretic medication was administered, (3) ICU stay was shorter than 6 hours after the detected temperature maximum, (4) documentation revealed an explicit indication for dipyrone or acetaminophen administration other than antipyresis, or (5) if active cooling devices (intravascular cooling devices or cooling blankets) were used during the study interval. Because we aimed to compare temperature courses of patients treated with either dipyrone or acetaminophen versus no medication, patients receiving both dipyrone and acetaminophen were excluded from primary endpoint analysis.

### Data collection

Paperless documentation on the study ICU is performed with the patient data management system (PDMS) m.life (medisite GmbH, Hannover, Germany). Patient data (anthropometric and baseline data, medication, medical history, vital parameters, ICU data) were extracted manually into a Microsoft Excel based ((Microsoft Corporation, Redmond, WA) study work sheet with this data being stored on a secured intrahospital server. After completion and cross-checking of these work sheets, identifying patient data were deleted and afterwards exported to SPSS (SPSS, Chicago, IL) for anonymized data analyses. In all patients, maximum and minimum documented body temperatures in the 24h time intervals prior to and after antipyretic treatment or temperature maximum were recorded.

### Study endpoints and main outcome measures

To compare effectiveness of each dipyrone and acetaminophen versus temperature courses without medication, we defined three main study groups. We analyzed temperature courses within 8 hours after administration of either dipyrone or acetaminophen. These temperature courses were compared to an untreated control group of patients starting with the temperature maximum. Additionally, we planned to explore antipyretic effectiveness of dipyrone and acetaminophen in the subgroup of patients with a body temperature of at least 38.0°C in order to adopt the HEAT trial definition of fever [12]. Individual patient charts were screened for the documented dipyrone and acetaminophen indications. To detect differences in antipyretic effectiveness of dipyrone and acetaminophen in comparison to an untreated control group, we defined maximum temperature change within 8 hours after antipyretic treatment (or temperature maximum in untreated patients) as the primary endpoint. Secondary endpoints were (1) length of ICU stay, (2) acute kidney injuries with their respective stages according to KDIGO criteria, and (3) ICU mortality [19].

### Statistical analysis

Because of the explorative design of this study, we aimed to include the largest sample size possible to obtain from the study ICU database. According to the HEAT trial, antipyretic effectiveness of acetaminophen exceeded placebo by 0.3°C (standard deviation of 1.0°C in the placebo group) [12]. We therefore defined 0.3°C as the minimal clinically relevant difference. With a global alpha of 0.05 and a power of 0.9, we calculated a total sample size of 604 patients to detect this difference comparing three subgroups of patients (no medication, only dipyrone, only acetaminophen) based on parametric testing. Adding 15% of cases for non-parametric testing and another 10% for missing values, we aimed at a sample size of at least 765 patients (255 patients per group) for the final analysis dataset. We were only able to extend the maximum recruitment interval back to January 2015 because of database consistency reasons and to avoid inference with the database of a previously published study [16].

Data are presented as medians with their respective interquartile range (IQR) or as means and 95% confidence interval (CI) as appropriate. Data were tested non-parametrically, because temperature study data are distributed non-normally for methodological reasons. We used Mann-Whitney test for two group comparisons and Kruskal-Wallis test for comparisons of three or more subgroups of unpaired data. Friedman test was used to compare subgroups with paired data (i.e. temperature data over time). Global level of significance was set at a p-value of less than 0.05. Bonferroni-Holm correction was used for subgroup comparisons [20]. Patients with missing temperature data were excluded from analysis of only the respective time point. Data were analysed with SPSS (SPSS 28.0, Chicago, Illinois, USA) and MedCalc (MedCalc 20.009, Ostende, Belgium).

## Results

### Patient characteristics

**Fig 1:**
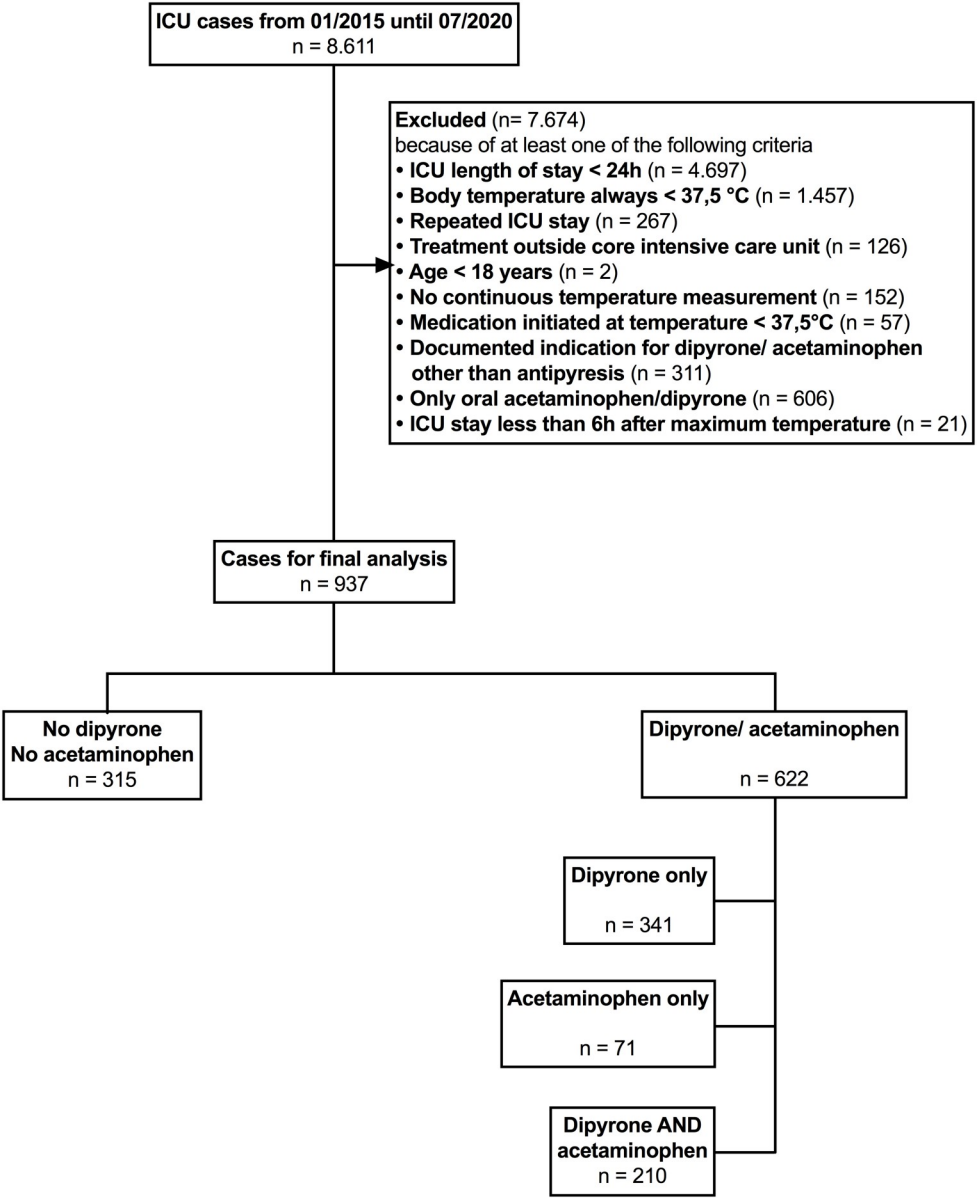
Patient flow chart. Study flowchart showing the number of patients and respective exclusion criteria. Patients receiving both dipyrone and acetaminophen were excluded from primary endpoint analysis for methodical reasons, but remained in the study for further analyses.

From January 2015 until July 2020, all 8611 admissions to the ICU were screened for this study (Fig 1). After verification of inclusion and exclusion criteria, 937 patients remained for the final analysis dataset (Fig 1). Of these, 504 (53.8%) patients were admitted to the ICU perioperatively, and 433 (46.2%) patients were admitted for medical or neurological health disorders. Baseline characteristics of the patients are shown in Table 1. The acetaminophen subgroup (n = 71) included into this study was smaller than planned, but the dipyrone subgroup (n = 341) and control group (n = 315) reached the targeted sample sizes.

**Table 1:** Baseline characteristics of the patients.

Quantitative Parameters	Total (n = 937)	No Med (n = 315)	Dipyrone only (n = 341)	Acetaminophen only (n = 71)	
	Median (IQR)	Median (IQR)	Median (IQR)	Median (IQR)	p-value
Age (y)	67 (54 to 77)	71 (59 to 80)	67 (54 to 76)	62 (54 to 78)	**0.002**
Weight (kg)	80 (68 to 90)	76 (65 to 90)	79 (65 to 90)	80 (70 to 90)	0.302
Height (cm)	172 (165 to 180)	170 (165 to 179)	173 (165 to 180)	175 (165 to 180)	0.019
SAPS-II at admission	40 (31 to 49)	40 (33 to 50)	39 (30 to 48)	41 (30 to 52)	0.078
Serum-Creatinine (μmol/L)	101 (83 to 131)	109 (88 to 135)	97 (82 to 122)	97 (82 to 142)	0.107
**Qualitative Parameters**	Total (n = 937)	No Med (n = 315)	Dipyrone (n = 341)	Acetaminophen (n = 71)	
	% (n)	% (n)	% (n)	% (n)	p-value
**Gender male**	57.2 (536)	52.7 (166)	58.9 (201)	60.6 (43)	0.207
**Ventilated at admission**	55.2 (517)	47.0 (148)	47.8 (163)	57.8 (41)	0.221
**Admission b/o Stroke**	16.9 (158)	16.2 (51)	16.7 (57)	16.9 (12)	0.979
**Admission b/o ICB**	5.8 (54)	4.8 (15)	4.7 (16)	5.6 (4)	0.943
**CAD**	18.6 (174)	24.4 (77)	15.8 (54)	19.7 (14)	**0.015**
**CHF**	14.9 (140)	18.7 (59)	11.4 (39)	15.5 (11)	**<0.001**
**Stroke**	13.8 (129)	14.3 (45)	12.3 (42)	14.1 (10)	0.317
**CKD**	16.6 (156)	21.6 (68)	14.7 (50)	15.5 (11)	0.057
**COPD**	10.4 (97)	11.7 (37)	9.4 (32)	11.3 (8)	0.221
**Diabetes**	18.2 (171)	20.6 (65)	15.2 (52)	15.5 (11)	0.089
**Sepsis**	21.6 (202)	17.8 (56)	17.3 (59)	23.9 (17)	0.612

CAD, coronary heart disease; CHF, chronic heart failure; CKD, chronic kidney disease; COPD, chronic obstructive pulmonary disease; ICB, intracranial bleeding; IQR, interquartile range; SAPS, simplified acute physiology score. p-value: Kruskal-Wallis H test for the three presented subgroups. Patients receiving both dipyrone and acetaminophen are included in the total column, but excluded from the dipyrone and acetaminophen columns. The acetaminophen group was considerably smaller than the dipyrone group. Patients receiving acetaminophen only were younger than patients receiving dipyrone only or no medication. Postoperatively admitted patients more likely received dipyrone than acetaminophen. Patients receiving dipyrone only less likely suffered from coronary artery disease or chronic heart failure than patients receiving no medication or acetaminophen only.

### Median body temperature decreased in all patient groups

**Fig 2:**
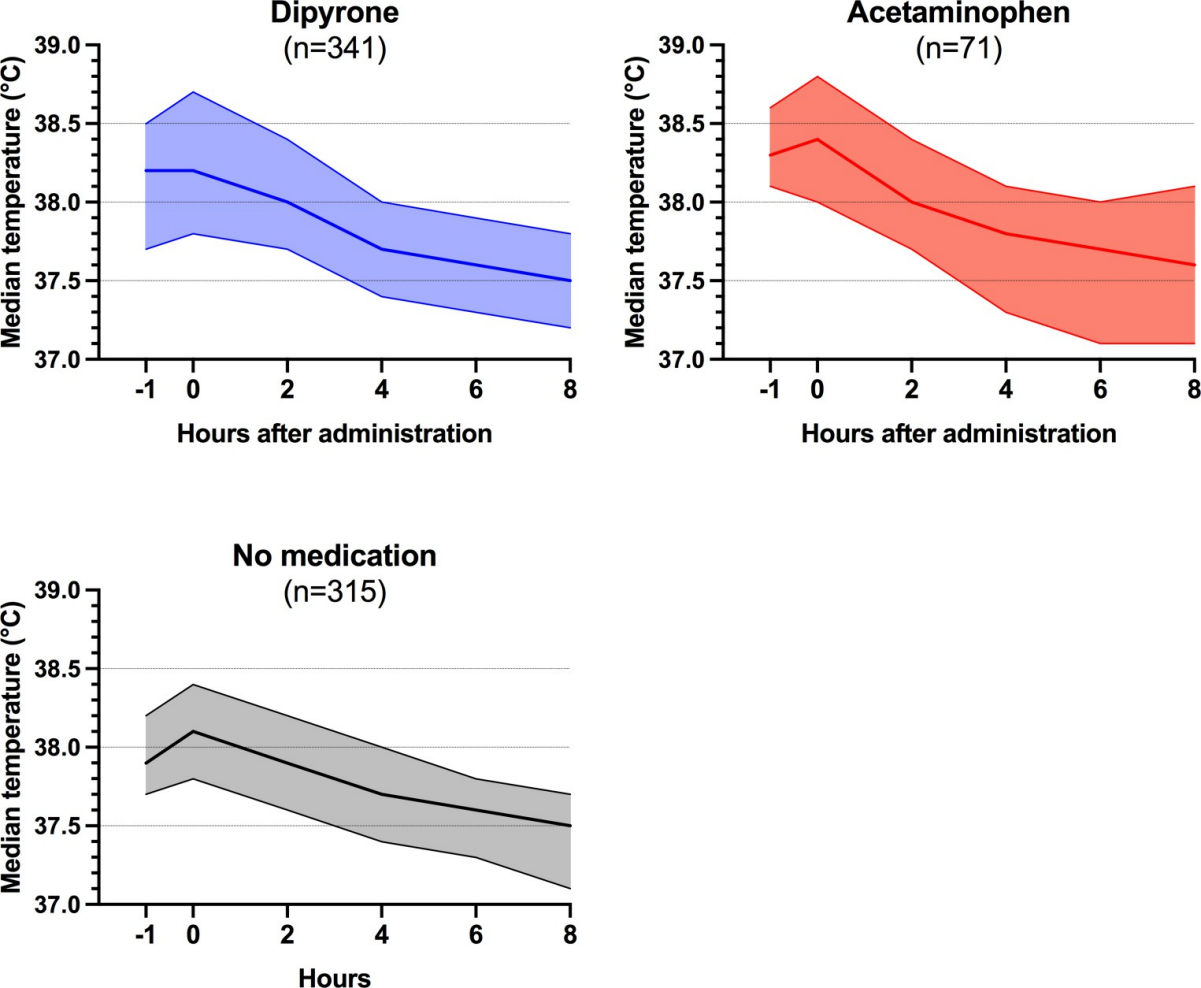
Median body temperature trends over time. Patients receiving either dipyrone (blue) or acetaminophen (red) and patients receiving no antipyretic treatment (grey) are shown separately. Median body temperatures with their respective 95% confidence interval are shown. Each four patients in the no medication and dipyrone group were excluded from the eight-hour time point because of missing temperature data. Patients receiving both dipyrone and acetaminophen are not shown. All patient groups presented with a decreasing median body temperature within the eight-hour study interval (Friedman test p<0.001; Friedman test for each of the three subgroups without correction for multiple testing p<0.001).

Median body temperature of all included patients was 38.3°C (IQR 37.9–38.6°C) at study inclusion and marginally differed among the groups (median: dipyrone 38.2°C (IQR 37.8–38.7°C), acetaminophen 38.4°C (IQR 38.0–38.8°C), no medication 38.1°C (IQR 37.8–38.4°C); p <0.001). Maximum temperature within 24 hours prior to the study interval also differed among the groups, but with marginal clinical significance (median: dipyrone 38.3°C (IQR 37.9–38.8°C), acetaminophen 38.5°C (IQR 38.3–38.8°C), no medication 38.1°C (IQR 37.8–38.4°C); p <0.001). Patient groups receiving dipyrone, acetaminophen or no antipyretic treatment each presented with a median decrease in body temperature (Friedman test for each dipyrone, acetaminophen and no medication group p<0.001, Fig 2). Lowest temperature within 24 hours after first antipyretic medication did not differ significantly between the groups (median: dipyrone 37.0°C (IQR 36.7–37.4°C), acetaminophen 37.0°C (IQR 36.6–37.4°C), no medication 37.0°C (IQR 36.6–37.4°C); p = 0.451).

### Primary endpoint

**Fig 3:**
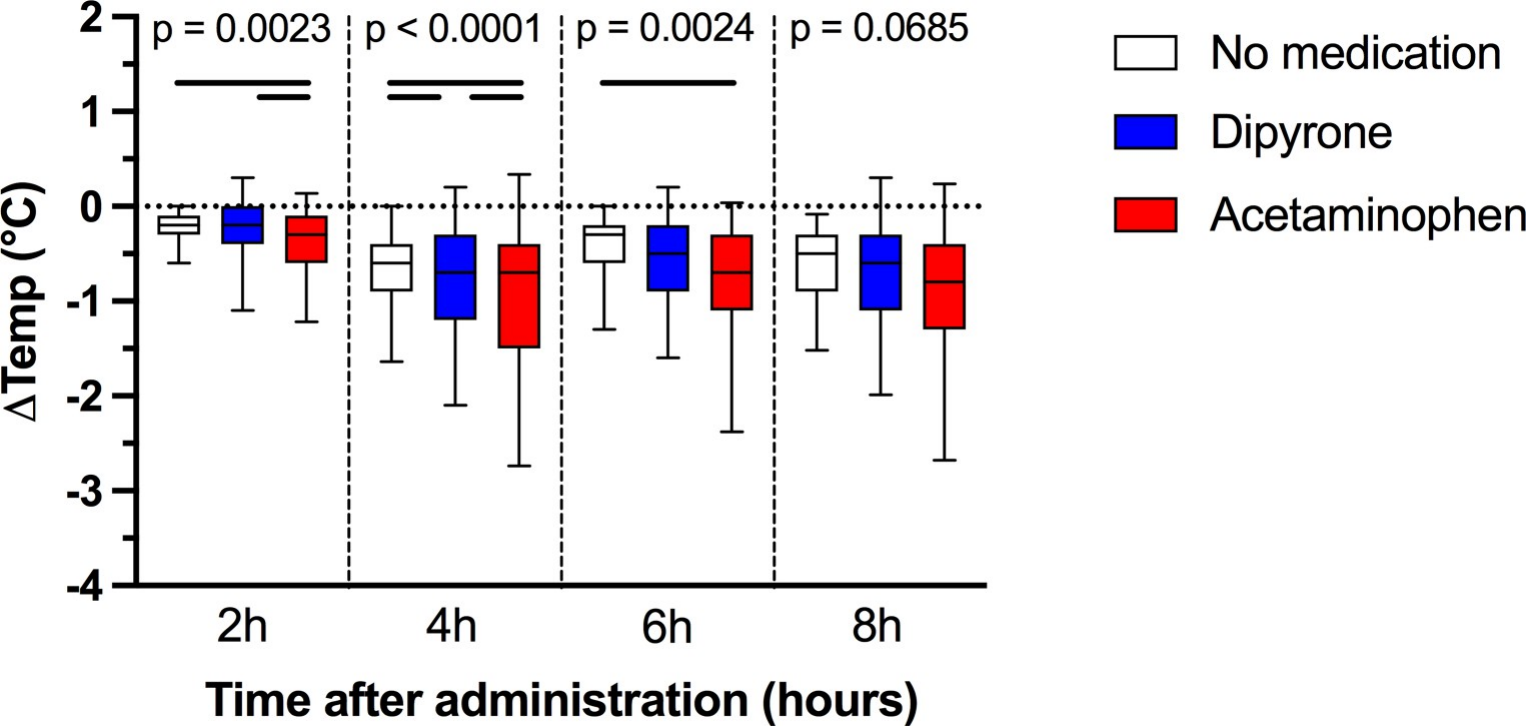
Maximum body temperature decrease within eight hours after administration of dipyrone, acetaminophen or no medication. Patients are grouped according to receiving either dipyrone (blue; n = 341) or acetaminophen (red; n = 71) versus patients receiving no antipyretic treatment (n = 315). P values are uncorrected Kruskal-Wallis p values for each time point. Black lines indicate significant differences (p <0.05, corrected for multiple testing). Two hours after administration, acetaminophen was associated with a higher decrease in body temperature than dipyrone or no medication. Dipyrone led to a higher temperature decrease than no medication starting 4 hours after administration.

**Fig 4:**
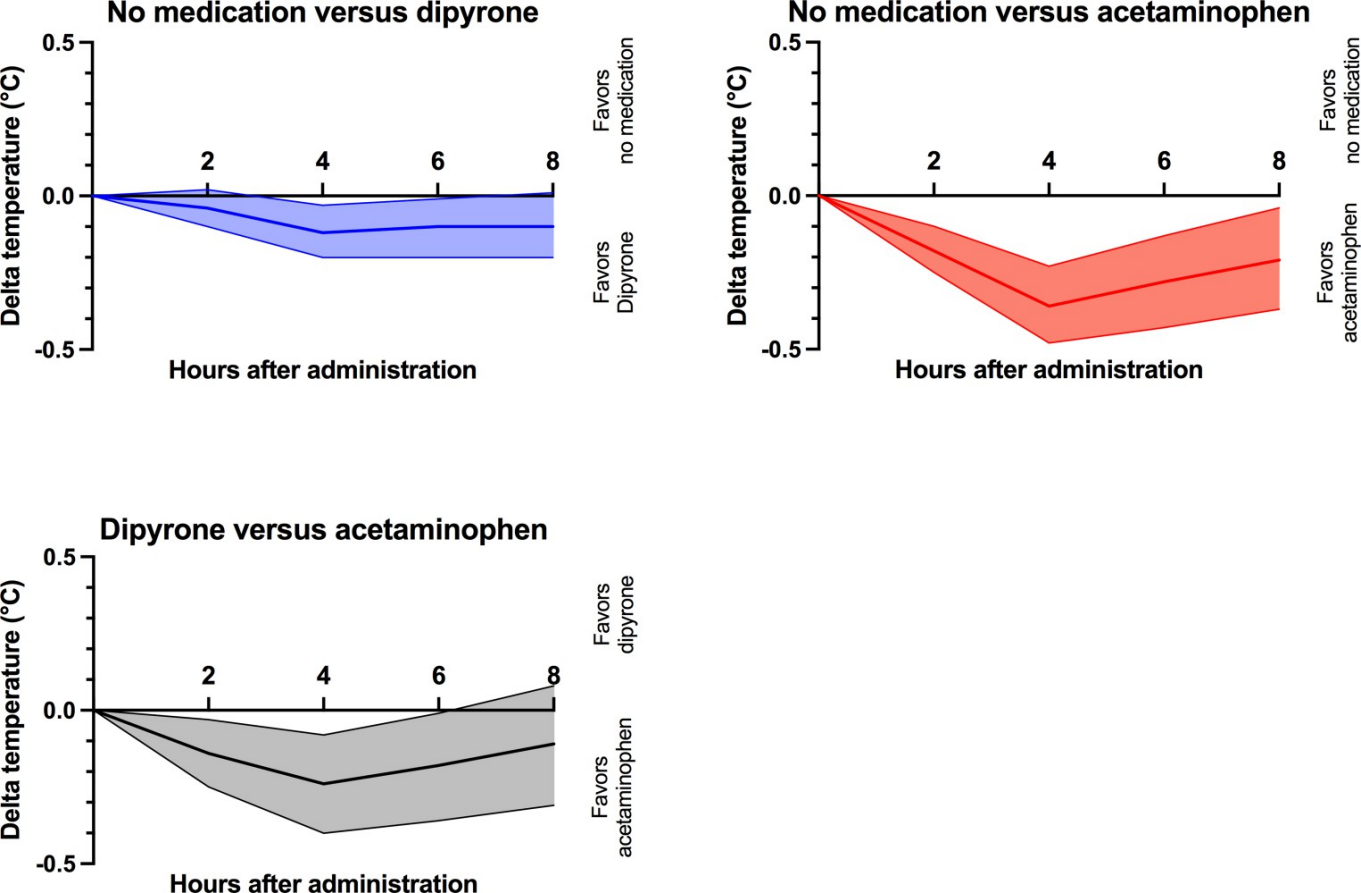
Comparison of mean differences in temperature decreases. Patients are grouped according to receiving either dipyrone (blue; n = 341) or acetaminophen (red; n = 71) versus patients receiving no antipyretic treatment (n = 315). Graphs display mean differences in temperature decreases between each two comparators (no medication versus dipyrone, no medication versus acetaminophen and dipyrone versus acetaminophen) with their respective 95% confidence intervals. Maximum of temperature decrease in association with dipyrone or acetaminophen was detected four hours after administration. Afterwards, body temperatures converged independent from the treatment group. Acetaminophen was associated with a steeper and more prolonged temperature decrease than dipyrone.

During the eight-hour study interval, median body temperature dropped to 37.5°C (IQR 37.2–37.9°C). Acetaminophen was associated with a more pronounced decrease in body temperature than dipyrone at two (p = 0.013) and four (p = 0.017) hours after administration, but temperature decreases six and eight hours after administration between dipyrone and acetaminophen treated patients did not significantly differ (p = 0.237; Fig 3). To visualize the effects of dipyrone and acetaminophen versus no antipyretic medication, mean differences in temperature changes were plotted (i.e. median temperature decrease of dipyrone/ acetaminophen minus temperature decrease in untreated patients; Fig 4). Two hours after treatment and compared to no medication, dipyrone was not associated with a lower body temperature (-0.2°C (IQR: -0.4 to 0.0°C); p = 0.802), while acetaminophen was associated with a higher decrease in body temperature (-0.3°C (IQR: -0.6 to -0.1°C) versus -0.2°C (IQR: -0.3 to -0.1); p = 0.001). Four hours after treatment, maximum temperature decrease was reached both with dipyrone (delta -0.12°C (95% CI: -0.04 to -0.20°C; p = 0.006) and acetaminophen (delta -0.36°C (95% CI: -0.23 to -0.48°C; p<0.001). Eight hours after administration, we did not find a different mean decrease in body temperatures among the three groups (p = 0.069). Detailed temperature data can be found in S1 Table in S1 File.

During the eight-hour study interval, maximum body temperature delta in patients without antipyretic medication was -0.6°C (IQR: -1.0 to -0.4°C). In comparison to these untreated patients, maximal decrease in body temperature was higher both with dipyrone (-0.8°C (IQR: -1.2 to -0.4°C); p = 0.016) and acetaminophen (-0.9°C (IQR: -1.6 to -0.6°C); p<0.001). Within the eight-hour study interval, we did not find a different maximum temperature decrease with dipyrone versus acetaminophen (p = 0.066).

### Temperature trends in patients with fever

We additionally analyzed the subgroup of patients presenting with a body temperature of at least 38.0°C. Figures for this subgroup of patients can be found in the Additional file 1 (S2 Fig in S1 File). 142 patients without antipyretic treatment, 214 patients receiving dipyrone only and 57 patients receiving acetaminophen only could be included into this subgroup analysis. Temperature patterns were comparable to data found in the whole cohort with a maximum of median temperature decrease four hours after administration of antipyretic medication (S3 and S4 Figs in S1 File). Two hours after administration, dipyrone decreased body temperature -0.1°C (95% CI: 0.0 to -0.2°C; p = 0.001) and acetaminophen decreased body temperature -0.2°C (95% CI: -0.1 to -0.3°C; p<0.001) more than no medication. Four hours after administration, dipyrone decreased body temperature -0.2°C (95% CI: -0.1 to -0.3°C; p < 0.001) and acetaminophen decreased body temperature -0.3°C (95% CI: -0.1 to -0.5°C; p = 0.001) more than no medication. While acetaminophen seemed to achieve higher temperature decreases over time in a visual analysis (S4 Fig and S5 Table in S1 File), the antipyretic effectiveness of dipyrone and acetaminophen was not statistically significant at any time point.

### Secondary endpoints

Median duration of ICU care was 3.8 days (IQR: 1.9 to 10.4 days) with a median SAPS-II score of 40 (IQR: 31 to 49) at admission. SAPS-II scores at admission did not differ among treatment groups (p = 0.128). 43 (4.6%) patients were dependent on chronic renal replacement therapy. A total of 352 (37.6%) patients suffered an AKI as diagnosed by the KDIGO criteria, including 272 (29.0%) patients with AKI stage 1, 55 (5.9%) with AKI stage 2 and 25 (2.7%) with AKI stage 3. No differences in AKI incidence were detected in association with antipyretic treatment (p = 0.151).

121 (12.9%) patients died during ICU treatment and mortality was not different among the antipyretic treatment groups (p = 0.263).

## Discussion

The main findings of our retrospective study are that

1. temperature decline in dipyrone treated patients was only -0.1 to -0.2°C higher than in untreated critically ill patients,
2. median absolute temperature decline in dipyrone treated patients was -0.8°C within eight hours after administration in critically ill patients, and
3. the maximum of antipyretic effectiveness was reached in median four hours after administration.

Therefore, this retrospective study data suggest that antipyretic effectiveness of dipyrone in critically ill patients may be overestimated. Additionally, our study at least doubles cumulative sample size of previous studies and adds to current evidence due to inclusion of a representative spectrum of mixed surgical critically ill patients. Previous observational studies [13, 14] and a small randomized trial [21] studying the antipyretic effect of dipyrone all showed a maximal temperature decline of about -1.0°C comparable to the effect size of our study, but did not compare results to an untreated control group. Previous studies included nonsurgical and aneurysmal subarachnoid hemorrhage patients, thereby limiting generalization of findings to a postoperative surgical patient collective. The findings of this study therefore emphasize that it is of utmost importance to compare antipyretic effectiveness of dipyrone versus spontaneous temperature declines in critically ill patients.

Dipyrone and acetaminophen both are regularly used drugs for antipyretic treatment in critically ill patients in Germany [7]. Although it is contraindicated in hemodynamically unstable patients according to the manufacturer’s approved product information, it is regularly used for treatment of fever in septic patients in Germany [22].

In contrast to the common use of acetaminophen, effectiveness data for treatment of fever with dipyrone are lacking. We therefore conducted this retrospective study to estimate the antipyretic effectiveness of dipyrone. We included all patients with elevated temperature readings as defined by a cutoff ≥ 37.5°C, but also analyzed the subgroup of patients with fever (≥ 38.0°C). Because there is no universally accepted fever definition, we decided to use the fever definition of the HEAT trial (≥ 38.0°C) that evaluated antipyretic effectiveness of acetaminophen. The HEAT trial found an additional acetaminophen antipyretic effectiveness of only -0.3°C, although prior observational studies without an untreated control group suggested a temperature effect of around -0.8 to -1.0°C [13, 23]. To estimate the external validity of our data, we compared the effect of acetaminophen against an untreated control group in our cohort to the effect of acetaminophen in the HEAT trial. The fact that maximal temperature effect of acetaminophen in both studies is around -0.3°C strengthens the validity of our retrospective approach.

Regarding the explorative secondary clinical outcomes, we did not find an association of antipyretic treatment strategy (dipyrone, acetaminophen or no antipyretic medication) with the incidence of acute kidney injury, ICU length of stay or ICU mortality.

Due to retrospective design of this study, several limitations have to be acknowledged. We carefully defined a reproducible control group of ICU patients presenting with elevated body temperature but without antipyretic treatment. Core definition criteria were temperature kinetics with peak temperature measurements of at least 37.5°C (or 38.0°C in the fever subgroup). Clinical reasoning for or against antipyretic treatment in the presented study collective was not guided by a defined treatment standard and therefore is likely to contain uncontrolled bias. Choosing maximum temperature as the reference point in untreated patients, we by design calculated the most distinct decrease in body temperature in these untreated patients. This in theory might lead to an underestimation of the antipyretic effectiveness of dipyrone and acetaminophen within this study. However, findings of our study reproduced the antipyretic effectiveness of acetaminophen known from the prospective HEAT trial.

Our study was conducted in a monocentric design and data extraction was based on an automated and paperless patient data management system. While the availability of automated temperature readings increased data density as compared to manual documentation, single temperature readings might be erroneous. We validated data by four-eyes principle, but cannot completely exclude inaccurate temperature readings.

Documentation time of dipyrone or acetaminophen administration is partly user-dependent. While drug administrations in the ICU have to be documented promptly by procedural requirements, data time entries may be inaccurate within the range of minutes. We therefore restricted our analyses to representative bi-hourly temperatures. Additionally, we excluded patients with only oral antipyretic treatment from the analyses as the time point of ingestion as well as pharmacokinetics and pharmacodynamics might remarkably differ from intravenous medication.

Our study is strengthened by high primary data quality due to extraction from a PDMS. Including only critically ill patients with an ICU stay of at least 24 hours focused our analysis on clinically relevant hyperthermic and febrile episodes rather and reduced bias by perioperative temperature management problems due to a systematic exclusion of intermediate care cases. Additionally, we included a mixed collective of critically ill surgical patients representative for a postoperative surgical ICU and therefore propose a higher robustness of dipyrone antipyretic effectiveness than previous studies. Findings from this study parallelize findings for fever treatment using acetaminophen in critically ill patients. While observational data for acetaminophen effectiveness suggested a temperature decline around -0.8 to -1.0°C, prospective data from the HEAT trial with an adequately designed control group found much smaller effectiveness. Due to the retrospective nature of our study, findings should be interpreted with caution and judged as only hypothesis-generating. Evidence-based recommendations for antipyretic treatment of critically ill patients using acetaminophen were down-graded after the HEAT trial due to a worse benefit-to-risk balance [24]. Assuming that dipyrone does not exceed acetaminophen antipyretic effectiveness, a confirmative randomized trial for dipyrone would change current clinical practice and likely limit the use of dipyrone in critically ill patients.

## Conclusion

Antipyretic effectiveness of dipyrone in ICU patients likely is overestimated and may not exceed spontaneous temperature decreases by more than -0.1 to -0.2°C. While dipyrone was associated with a decrease in temperature of in median -0.8°C within eight hours after administration, patients without antipyretic medication also presented with a comparable pattern of spontaneous body temperature decrease within this time interval. The maximum of dipyrone antipyretic effectiveness was found four hours after intravenous administration. To our knowledge, this is the first and largest study investigating antipyretic effectiveness of dipyrone in a mixed collective of critically ill surgical patients. However, clinical evidence supporting antipyretic dipyrone therapy in the ICU is insufficient and warrants further prospective evaluation.

## Supporting information

S1 File (PDF)
