# Supplementary material for: The antipyretic effectiveness of dipyrone in the intensive care unit: A retrospective cohort study
Source: PLoS One. 2022 Mar 10;17(3):e0264440. doi: 10.1371/journal.pone.0264440 (PMC8912151; doi:10.1371/journal.pone.0264440)
Supplement: S1 File — (PDF) [file pone.0264440.s001.pdf]

Supplemental data

The antipyretic effectiveness of dipyrone in the intensive care unit: a retrospective cohort study

Hans-Jörg Gillmann[1]*, Jessica Reichart[1], Andreas Leffler[1], Thomas Stueber[1]

[1]Department of Anaesthesiology and Intensive Care Medicine, Hannover Medical School, OE 8050, Carl-Neuberg-Str. 1, D-30625 Hannover, Germany

***Corresponding author:**
E-mail: gillmann.hans-joerg@mh-hannover.de

Short title: Antipyretic effect of dipyrone in the ICU

Supporting information

S1 Table. Temperature data for the primary endpoint							
	Variable	N	Mean	SD	Percentile		
					25.	50. (Median)	75.
All groups	Tmin 24h before	937	36.9	1.0	36.3	36.9	37.5
	Tmax 24h before	937	38.7	11.1	37.9	38.3	38.7
	T at -1h	937	38.2	0.6	37.7	38.1	38.5
	T at 0h	937	38.3	0.5	37.9	38.3	38.6
	T at 2h	937	38.0	0.5	37.7	38.0	38.4
	T at 4h	937	37.8	0.6	37.4	37.7	38.1
	T at 6h	937	37.6	0.6	37.3	37.6	37.9
	T at 8h	927	37.6	0.7	37.2	37.5	37.9
	Tmax 24h after	937	38.4	0.6	38.0	38.4	38.8
	Tmin 24h after	937	37.0	0.6	36.6	37.1	37.4
	Tdiff 0-2h	937	-0.3	0.4	-0.4	-0.2	0.0
	Tdiff 0-4h	937	-0.6	0.6	-0.8	-0.4	-0.2
	Tdiff 0-6h	937	-0.7	0.7	-1.0	-0.6	-0.3
	Tdiff 0-8h	937	-0.8	0.7	-1.1	-0.7	-0.3
	Tdiff 0-4h max	937	-0.6	0.6	-0.8	-0.5	-0.2
	Tdiff 0-6h max	937	-0.8	0.6	-1.1	-0.6	-0.3
	Tdiff 0-8h max	937	-0.9	0.7	-1.2	-0.8	-0.5
No antipyretic medication	Tmin 24h before	315	36.6	0.8	36.1	36.7	37.1
	Tmax 24h before	315	38.1	0.4	37.8	38.1	38.4
	T at -1h	315	38.0	0.4	37.7	37.9	38.2
	T at 0h	315	38.1	0.4	37.8	38.1	38.4
	T at 2h	315	37.9	0.4	37.6	37.9	38.2
	T at 4h	315	37.7	0.5	37.4	37.7	38.0
	T at 6h	315	37.5	0.5	37.3	37.6	37.8
	T at 8h	311	37.4	0.5	37.1	37.5	37.7
	Tmax 24h after	315	38.1	0.4	37.8	38.1	38.4
	Tmin 24h after	315	36.9	0.7	36.6	37.0	37.4
	Tdiff 0-2h	315	-0.2	0.2	-0.3	-0.2	-0.1
	Tdiff 0-4h	315	-0.4	0.4	-0.6	-0.3	-0.2
	Tdiff 0-6h	315	-0.6	0.5	-0.9	-0.5	-0.3
	Tdiff 0-8h	311	-0.7	0.6	-0.9	-0.6	-0.4
	Tdiff 0-4h max	315	-0.5	0.4	-0.6	-0.4	-0.2
	Tdiff 0-6h max	315	-0.7	0.5	-0.9	-0.5	-0.3
	Tdiff 0-8h max	315	-0.8	0.6	-1.0	-0.6	-0.4
Dipyrone only	Tmin 24h before	341	36.9	1.0	36.4	37.0	37.5
	Tmax 24h before	341	38.4	0.6	37.9	38.3	38.8
	T at -1h	341	38.2	0.6	37.7	38.2	38.5
	T at 0h	341	38.3	0.6	37.8	38.2	38.7
	T at 2h	341	38.1	0.6	37.7	38.0	38.4
	T at 4h	341	37.7	0.6	37.4	37.7	38.0
	T at 6h	341	37.6	0.6	37.3	37.6	37.9
	T at 8h	337	37.5	0.6	37.2	37.5	37.8
	Tmax 24h after	341	38.4	0.6	38.0	38.4	38.8
	Tmin 24h after	341	37.0	0.6	36.7	37.0	37.4
	Tdiff 0-2h	341	-0.3	0.4	-0.4	-0.2	0.0
	Tdiff 0-4h	341	-0.6	0.6	-0.9	-0.5	-0.2
	Tdiff 0-6h	341	-0.7	0.7	-1.1	-0.6	-0.3
	Tdiff 0-8h	337	-0.8	0.7	-1.2	-0.7	-0.3
	Tdiff 0-4h max	341	-0.6	0.6	-0.9	-0.5	-0.2
	Tdiff 0-6h max	341	-0.8	0.7	-1.1	-0.7	-0.3
	Tdiff 0-8h max	341	-0.9	0.7	-1.2	-0.8	-0.4

S1 Table. Temperature data for the primary endpoint (Continued)

	Variable	N	Mean	SD	Percentile		
					25.	50. (Median)	75.
**Acetaminophen only**	**Tmin 24h before**	210	37.2	1.0	36.4	37.2	37.8
	**Tmin 24h before**	71	36.9	0.9	36.2	37.0	37.6
	**Tmax 24h before**	71	38.6	0.5	38.3	38.5	38.8
	**T at -1h**	71	38.4	0.5	38.1	38.3	38.6
	**T at 0h**	71	38.4	0.6	38.0	38.4	38.8
	**T at 2h**	71	38.0	0.6	37.7	38.0	38.4
	**T at 4h**	71	37.6	0.7	37.3	37.8	38.1
	**T at 6h**	71	37.5	0.8	37.1	37.7	38.0
	**T at 8h**	71	37.5	0.8	37.1	37.6	38.1
	**Tmax 24h after**	71	38.6	0.5	38.2	38.5	38.9
	**Tmin 24h after**	71	36.9	0.7	36.6	37.0	37.4
	**Tdiff 0-2h**	71	-0.4	0.4	-0.6	-0.3	-0.1
	**Tdiff 0-4h**	71	-0.8	0.7	-1.1	-0.7	-0.3
	**Tdiff 0-6h**	71	-0.9	0.8	-1.3	-0.8	-0.4
	**Tdiff 0-8h**	71	-0.9	0.9	-1.5	-0.7	-0.4
	**Tdiff 0-4h max**	71	-0.8	0.6	-1.1	-0.8	-0.3
	**Tdiff 0-6h max**	71	-1.0	0.7	-1.4	-0.9	-0.5
	**Tdiff 0-8h max**	71	-1.1	0.8	-1.6	-0.9	-0.6
**Dipyrone AND Acetaminophen**	**Tmin 24h before**	210	37.2	1.0	36.4	37.2	37.8
	**Tmax 24h before**	210	39.3	0.5	38.3	38.6	39.0
	**T at -1h**	210	38.5	0.5	38.1	38.4	38.8
	**T at 0h**	210	38.6	0.5	38.2	38.5	38.9
	**T at 2h**	210	38.2	0.6	37.9	38.2	38.5
	**T at 4h**	210	37.9	0.7	37.5	37.9	38.4
	**T at 6h**	210	37.8	0.7	37.3	37.8	38.3
	**T at 8h**	208	37.8	0.7	37.3	37.7	38.3
	**Tmax 24h after**	210	38.8	0.6	38.4	38.7	39.1
	**Tmin 24h after**	210	37.1	0.7	36.7	37.2	37.6
	**Tdiff 0-2h**	210	-0.3	0.5	-0.5	-0.2	0.0
	**Tdiff 0-4h**	210	-0.6	0.7	-1.0	-0.5	-0.2
	**Tdiff 0-6h**	210	-0.7	0.8	-1.1	-0.6	-0.3
	**Tdiff 0-8h**	208	-0.7	0.8	-1.1	-0.7	-0.3
	**Tdiff 0-4h max**	210	-0.7	0.7	-1.0	-0.6	-0.3
	**Tdiff 0-6h max**	210	-0.8	0.7	-1.2	-0.7	-0.4
	**Tdiff 0-8h max**	210	-0.9	0.7	-1.2	-0.9	-0.5

Abbreviations: diff, difference; h, hour; max, maximum; min, minimum; N, patient number; SD, standard deviation; T, temperature. Four patients in the no medication and in the dipyrone group as well as two patients in the dipyrone and acetaminophen group had missing temperature data eight hours post administration and therefore had to be excluded from analysis of this timepoint.

S2 Fig. Median body temperature trends over time in patients with a minimum body temperature of 38.0°C

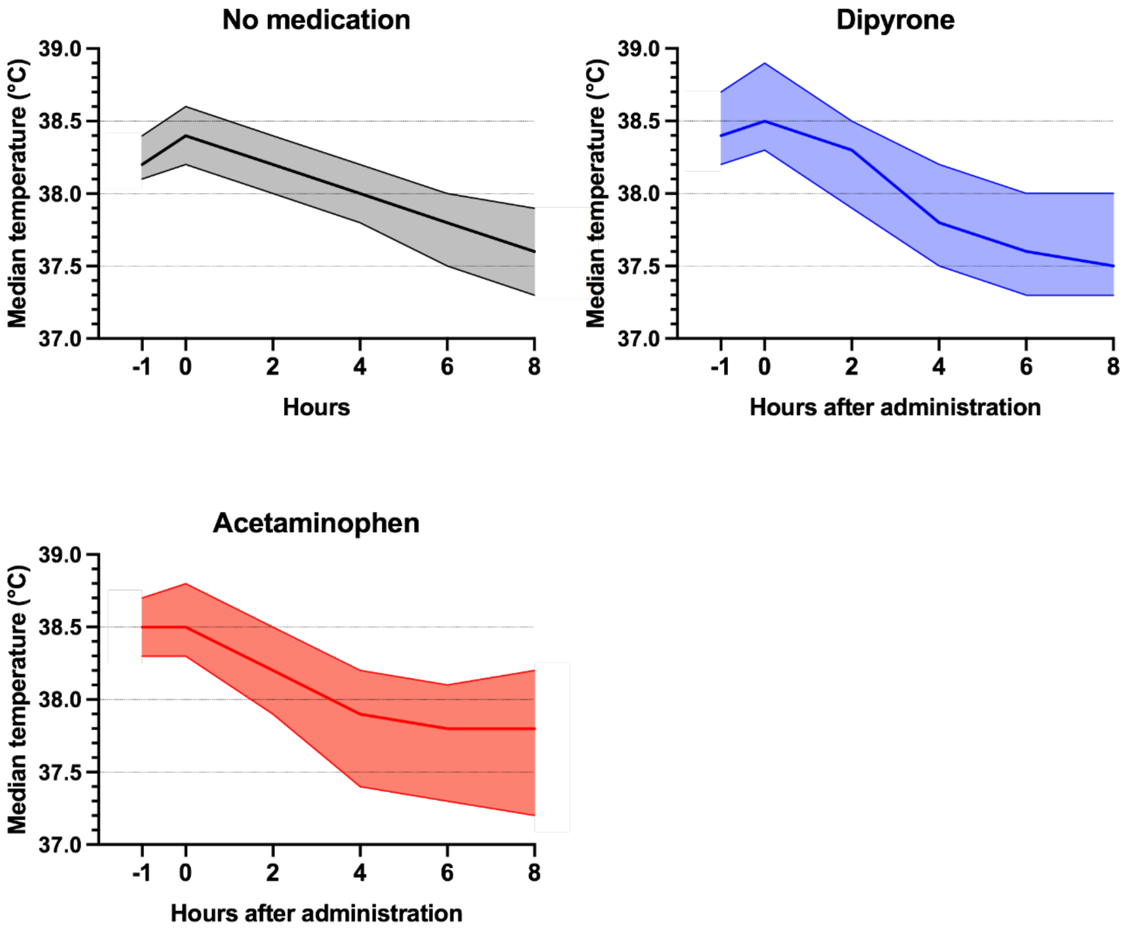

Patients receiving either dipyrone (blue) or acetaminophen (red) and patients receiving no antipyretic treatment (grey) are shown separately. Median body temperatures with their respective 95% confidence interval are shown. This subgroup summarizes patients presenting with a temperature of at least 38.0 °C at one hour prior to antipyretic treatment (i.e. the beginning of the shown graphs). The temperature one hour prior to antipyretic treatment was supposed to represent the temperature for clinical treatment decision. Three patients in the no medication and one patient in the dipyrone group were excluded from the eight hour time point because of missing temperature data. Patients receiving both dipyrone and acetaminophen are not shown. All patient groups presented with a decreasing median body temperature within the eight hour study interval (Friedman test $p<0.001$; Friedman test for each of the three subgroups without correction for multiple testing $p<0.001$).

S3 Fig. Comparison of mean differences in temperature decreases in patients with a minimum body temperature of 38.0°C

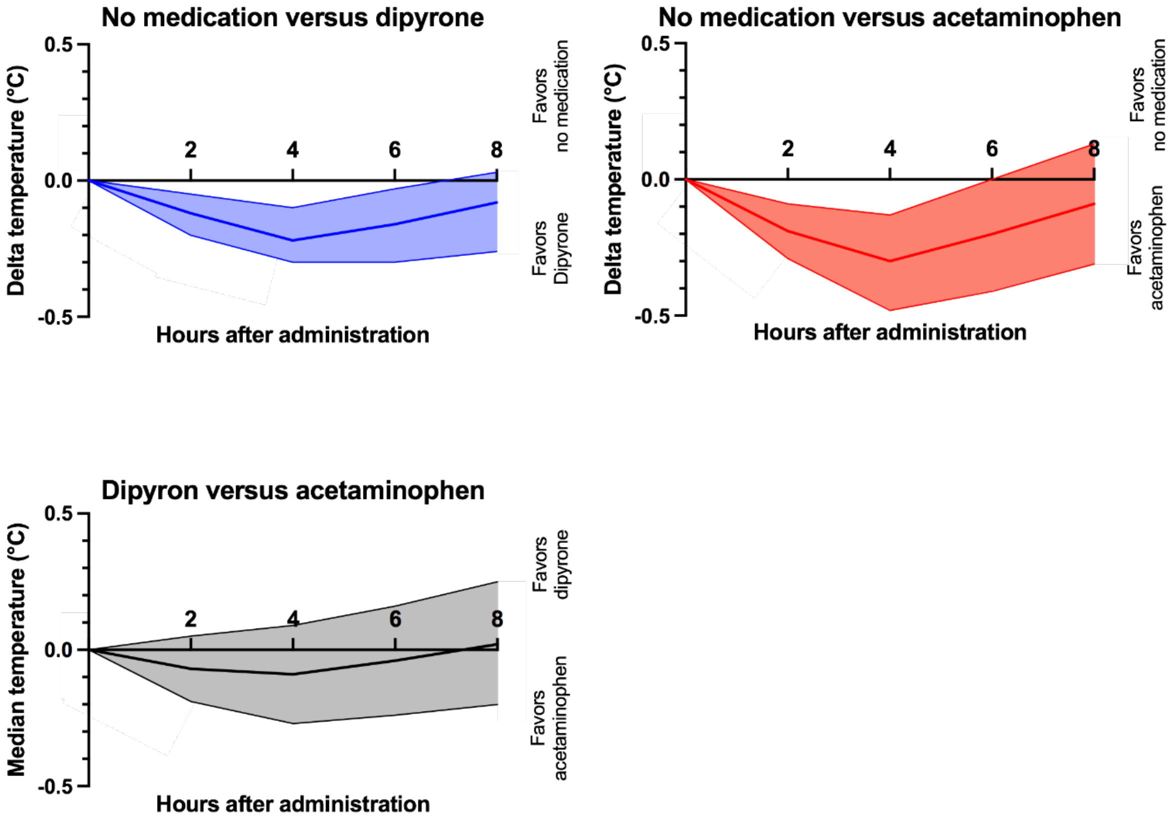

Patients receiving either dipyrone (blue) or acetaminophen (red) and patients receiving no antipyretic treatment (grey) are shown separately. Median body temperatures with their respective 95% confidence interval are shown. This subgroup summarizes patients presenting with a temperature of at least 38.0 °C at one hour prior to antipyretic treatment (i.e. the beginning of the shown graphs). The temperature one hour prior to antipyretic treatment was supposed to represent the temperature for clinical treatment decision. Three patients in the no medication and one patient in the dipyrone group were excluded from the eight hour time point because of missing temperature data. Patients receiving both dipyrone and acetaminophen are not shown. All patient groups presented with a decreasing median body temperature within the eight hour study interval (Friedman test $p<0.001$; Friedman test for each of the three subgroups without correction for multiple testing $p<0.001$). Confidence intervals in this patient subgroup were wider than for the whole cohort because auf the smaller patient number. Compared to no

antipyretic treatment, only acetaminophen led to a decrease in body temperature different from zero at four hours after administration.

S4 Fig. Antipyretic efficacy of dipyrone and acetaminophen during eight hours after administration

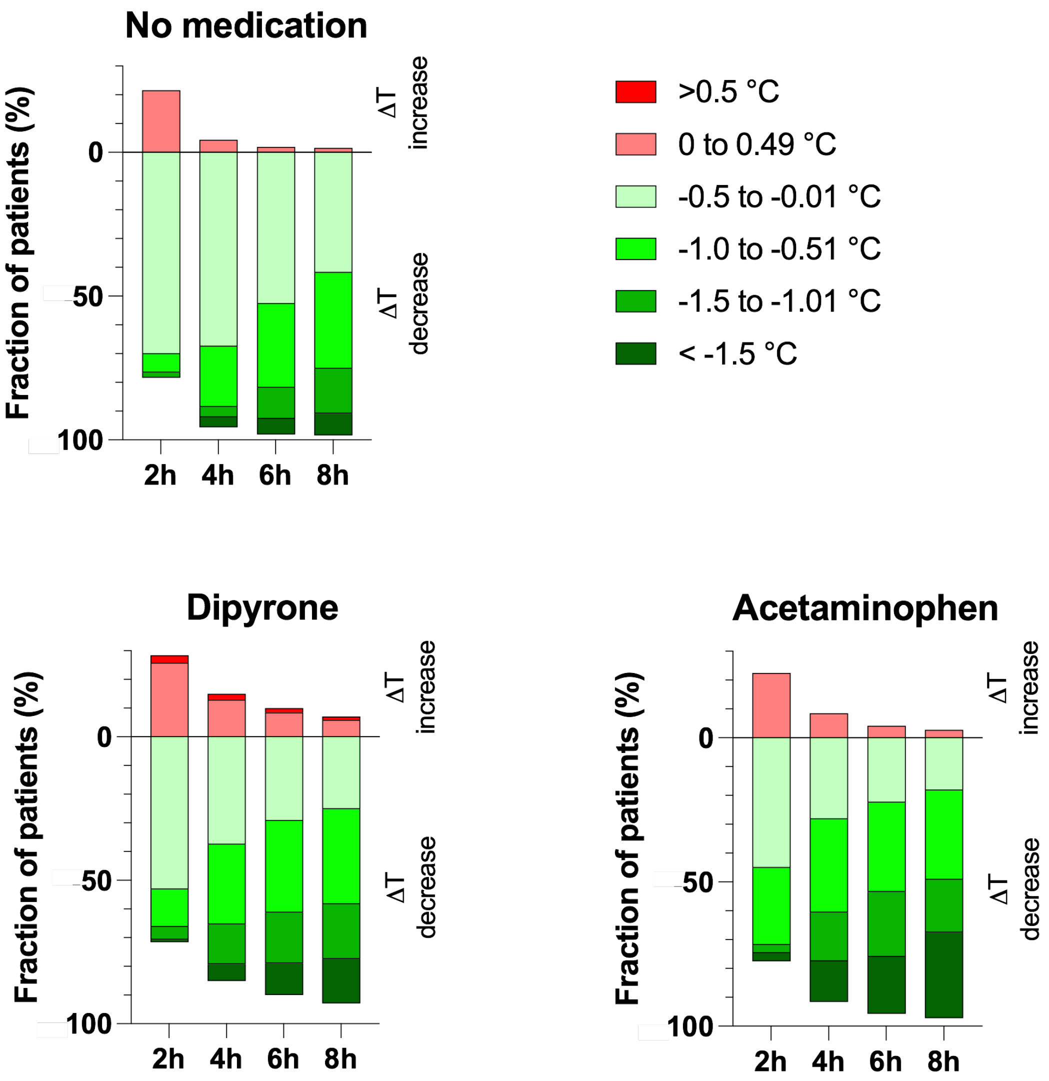

Patients receiving either dipyrone, acetaminophen or no antipyretic treatment are shown separately. Patients receiving both dipyrone and acetaminophen are not shown. Patients are grouped by 0.5°C steps of temperature decrease with 0h (administration of drug) as the reference. Patients with a temperature increase are coloured red, patients with a temperature decrease are coloured green. The more the temperature decreased during the study interval, the more darker green the bar gets. Acetaminophen led to a higher proportion of patients with body temperature decreases of more than 1.5 °C six and eight hours after administration in comparison to dipyrone, but this effect did not reach statistical significance ($p=0.358$ at six hours and $p=0.352$ at eight hours).

S5 Table. Cross tables for changes in body temperature over time by antipyretic treatment group

No Med/ 0h

2h	<37.5	37.5-37.99	38.0-38.49	38.5-39.99	40+	
<37.5	0	27	2	0	0	29
37.5-37.99	0	95	49	6	0	150
38.0-38.49	0	0	80	27	0	107
38.5-39.99	0	0	0	29	0	29
40+	0	0	0	0	0	0
	0	122	131	62	0	315
T increase	111	35,2 %				
T decrease	0	0 %				

No Med/ 0h

4h	<37.5	37.5-37.99	38.0-38.49	38.5-39.99	40+	
<37.5	0	57	19	8	0	84
37.5-37.99	0	65	68	13	0	146
38.0-38.49	0	0	44	30	0	74
38.5-39.99	0	0	0	11	0	11
40+	0	0	0	0	0	0
	0	122	131	62	0	315
T increase	195	61,9 %				
T decrease	0	0 %				

No Med/ 0h

6h	<37.5	37.5-37.99	38.0-38.49	38.5-39.99	40+	
<37.5	0	57	19	8	0	84
37.5-37.99	0	65	68	13	0	146
38.0-38.49	0	0	44	30	0	74
38.5-39.99	0	0	0	11	0	11
40+	0	0	0	0	0	0
	0	122	131	62	0	315
T increase	195	61,9 %				
T decrease	0	0 %				

No Med/ 0h

8h	<37.5	37.5-37.99	38.0-38.49	38.5-39.99	40+	
<37.5	0	82	43	16	0	141
37.5-37.99	0	36	68	29	0	133
38.0-38.49	0	1	19	14	0	34
38.5-39.99	0	0	0	3	0	3
40+	0	0	0	0	0	0
	0	119	130	62	0	311
T increase	252	81 %				
T decrease	1	0,3 %				

Dipyrone/ 0h

2h	<37.5	37.5-37.99	38.0-38.49	38.5-39.99	40+	
<37.5	0	31	8	2	0	41
37.5-37.99	0	68	40	13	0	121
38.0-38.49	0	15	51	43	0	109
38.5-39.99	0	2	4	60	2	68
40+	0	0	0	0	2	2
	0	116	103	118	4	341
T increase	139	40,8 %				
T decrease	21	6,2 %				

Dipyrone/ 0h

4h	<37.5	37.5-37.99	38.0-38.49	38.5-39.99	40+	
<37.5	0	48	28	15	0	91
37.5-37.99	0	56	51	46	1	154
38.0-38.49	0	10	21	34	0	65
38.5-39.99	0	2	3	23	2	30
40+	0	0	0	0	1	1
	0	116	103	118	4	341
T increase	225	66 %				
T decrease	15	4,4 %				

Dipyrone/ 0h

6h	<37.5	37.5-37.99	38.0-38.49	38.5-39.99	40+	
<37.5	0	59	39	32	1	131
37.5-37.99	0	50	42	47	0	139
38.0-38.49	0	5	17	26	1	49
38.5-39.99	0	2	5	12	1	20
40+	0	0	0	1	1	2
	0	116	103	118	4	341
T increase	248	72,7 %				
T decrease	13	3,8 %				

Dipyrone/ 0h

8h	<37.5	37.5-37.99	38.0-38.49	38.5-39.99	40+	
<37.5	0	66	45	39	1	151
37.5-37.99	0	44	36	40	1	121
38.0-38.49	0	5	12	26	0	43
38.5-39.99	0	0	7	13	1	21
40+	0	0	0	0	1	1
	0	115	100	118	4	337
T increase	255	75,7 %				
T decrease	12	3,6 %				

PCM/ 0h

2h	<37.5	37.5-37.99	38.0-38.49	38.5-39.99	40+	
<37.5	0	6	2	1	0	9
37.5-37.99	0	7	10	5	0	22
38.0-38.49	0	0	12	12	1	25
38.5-39.99	0	0	0	15	0	15
40+	0	0	0	0	0	0
	0	13	24	33	1	71
T increase	37	52,1 %				
T decrease	0	0 %				

PCM/ 0h

4h	<37.5	37.5-37.99	38.0-38.49	38.5-39.99	40+	
<37.5	0	10	8	7	0	25
37.5-37.99	0	3	9	5	1	18
38.0-38.49	0	0	7	18	0	25
38.5-39.99	0	0	0	3	0	3
40+	0	0	0	0	0	0
	0	13	24	33	1	71
T increase	58	81,7 %				
T decrease	0	0 %				

PCM/ 0h

6h	<37.5	37.5-37.99	38.0-38.49	38.5-39.99	40+	
<37.5	0	10	11	7	0	28
37.5-37.99	0	2	6	11	1	20
38.0-38.49	0	1	6	8	0	15
38.5-39.99	0	0	1	7	0	8
40+	0	0	0	0	0	0
	0	13	24	33	1	71
T increase	54	76,1 %				
T decrease	2	2,8 %				

PCM 0h

8h	<37.5	37.5-37.99	38.0-38.49	38.5-39.99	40+	
<37.5	0	9	12	10	0	31
37.5-37.99	0	2	6	10	1	19
38.0-38.49	0	2	5	7	0	14
38.5-39.99	0	0	1	6	0	7
40+	0	0	0	0	0	0
	0	13	24	33	1	71
T increase	55	77,5 %				
T decrease	3	4,2 %				

Tables show absolute numbers of patients grouped by body temperature at 0h (columns) and respective group at 2h, 4h, 6h and 8h after medication. Patients are subdivided into three groups: No medication, dipyrone only, acetaminophen only. Within the no medication group, 4 patients at 8h and 4 patients in the dipyrone group at 8h with missing body temperatures were excluded for this time point.
Body temperature is grouped in 0.5 °C cohorts: < 37.5°C, 37.5-37.99 °C, 38.0-38.49 °C, 38.5-39.99 °C, ≥ 40 °C. Increases in body temperature are coloured red, decreases in body temperature are coloured green. Patients without a change in body temperature are coloured grey.
Increases in body temperature after administration of dipyrone occured more frequently than after acetaminophen.
